# Unusual sex chromosomal DSD in a domestic Shorthair cat with a 37,X/38,XY mosaic karyotype

**DOI:** 10.1186/s12917-024-04164-y

**Published:** 2024-07-06

**Authors:** L. Montenegro, I. Costa, L. Maltez, V. Evaristo, I. R. Dias, C. Martins, I. Borges, F. Morinha, R. Pereira, N. Neto, C. Oliveira, A. Martins-Bessa

**Affiliations:** 1 Veterinary Hospital Referência Veterinária Montenegro, Porto, 4000-395 Portugal; 2 Animal and Veterinary Research Center (CECAV), University of Trás-os-Montes and Alto Douro (UTAD), Vila Real, 5000-801 Portugal; 3 AL4AnimalS-Associate Laboratory for Animal and Veterinary Sciences, UTAD, Vila Real, 5000-801 Portugal; 4 Canidelo Veterinary Clinic – OneVet Group, Vila Nova de Gaia, 4400-710 Portugal; 5 Cedivet, Lionessa Business Hub, Leça do Balio, 4465-671 Portugal; 6 Morinha Lab- Laboratory of Biodiversity and Molecular Genetics, Vila Real, 5000-562 Portugal; 7 Laboratory of Cell Biology, Department of Microscopy, ICBAS‑School of Medicine and Biomedical Sciences, University of Porto, Porto, 4050‑313 Portugal; 8 UMIB‑Unit for Multidisciplinary Research in Biomedicine, ICBAS‑UP/ ITR‑Laboratory for Integrative and Translational Research in Population Health, University of Porto, Porto, 4050‑313 Portugal; 9 Laboratory of Cytogenetics, Department of Microscopy, ICBAS‑School of Medicine and Biomedical Sciences, University of Porto, Porto, 4050‑313 Portugal

**Keywords:** Cat, Disorder of sexual development, Mosaicism, X/XY karyotype, Gonadal dysgenesis, *SRY*

## Abstract

**Background:**

Sex chromosome abnormalities associated with disorders of sexual development (DSD) are rarely described in cats, mainly due to the lack of chromossome studies that precisely reveal the condition. Genetic approaches are therefore required in order to detect sex chromossomes abnormalities as variations in the number and structure of chromosomes, or the presence of a second cell line as mosaicim or chimerism.

**Case presentation:**

A male Shorthair cryptorchid cat was presented with clinical signs of anorexia, tenesmus and hyperthermia. Ultrasonography revealed a fluid-filled structure, with approximately 1 cm in diameter, adjacent to the descending colon. Computed tomography evidenced a tubular structure, ventral to the descending colon and caudal to the bladder, which extended cranially, through two branches. Histopathological evaluation confirmed the presence of two atrophic uterine horns and one hypoplastic testicle with epididymis at the end of one of the uterine horns. The end of the other uterine horn was attached to a structure composed by a mass of adipocytes. Cytogenetic analysis revealed a mosaic 37,X/38,XY karyotype. The two cell lines were found in 15% and 85% of the lymphocytes, respectively. Genetic analysis confirmed the presence of *SRY* and *ZFY* genes in blood and hair bulbs, and revealed a marked reduction in *SRY* expression in the testicle. Additionally, this case presented exceptionally rare features, such as a Leydig’ cell tumour and a chronic endometritis in both uterine horns.

**Conclusions:**

Complete imaging workup, cytogenetic analysis and *SRY* gene expression should be systematically realized, in order to properly classify disorders of sexual development (DSD) in cats.

## Background

Disorders of sexual development (DSD), formerly defined by the term intersexuality or hermaphroditism, are important factors affecting reproduction due to deleterious effect on fertility, increased risk of gonadal cancerogenesis, behaviour problems, also often associated with other congenital malformations [1].

DSD are classified into three main categories: sex chromosomal DSD, caused by abnormalities of the sex chromosomes; XX DSD, when the individual with this disorder have a normal female sex chromosome complement, and XY DSD, to describe individuals with this disorder that have a normal male sex chromosome complement [1, 2].

On the other hand, abnormal internal genitalia with persistent Müllerian derivatives reveal failure of these structures to regress. These ducts give rise to the uterine tube, uterus and cranial vagina in females. This anomaly is the result of a deficiency in anti-Müllerian hormone or a lack of its receptor [3] and is associated, in approximately 50% of the cases, with cryptorchidism [4, 5]. This condition is well described in dogs, especially in the Miniature Schnauzer breed, where it is inherited as an autosomal recessive trait [5], but more rarely described in cat.

Animals with a DSD condition may not be diagnosed until they present clinical signs of pyometra, urinary tract infection, prostate disease or fever [5, 6].

Cytogenetic analysis of animals with abnormal genitalia has provided evidence that this phenotype is quite often associated with sex chromosome abnormalities in either the chromosome structure or their number, such as X monosomy [1]. However, there are very few reports of cats with a mosaic 37,X/38,XY karyotype [2, 7]. In these works, the cell lines 37,X/38,XY were found in 90% and 10%; 96% and 4% of the lymphocytes, respectively.

The aim of the present work is to describe a DSD in cat with 37,X/38,XY mosaic karyotype that presented very rare histopathological features. To the best of the authors’ knowledge, this is the first case reported in which a Leydig cell’s tumour and chronic endometritis are simultaneously associated in a cat with sex chromosomal DSD.

## Case presentation

### Clinical findings

A 3-year-old Domestic Shorthair cat weighing 4 kg was referred to the Hospital Referência Veterinária Montenegro (Oporto, Portugal) because of tenesmus, anorexia, vomiting and hyperthermia. The cat had a previous clinical history of obstipation, which was resolved with enemas.

Physical and genital examination revealed a large amount of hard stool in the colon and the absence of penile spines, as well as the absence of both testes in the scrotum. The current owner had rescued the cat from a colony and had no information about its past or whether the male had been neutered previously. According to the owners, the cat did not show male behaviour (aggression or marking behaviour) at home. No abnormal findings were found on complete blood cell count and serum biochemistry panel.

### Ultrasonographic findings

**Fig. 1:**
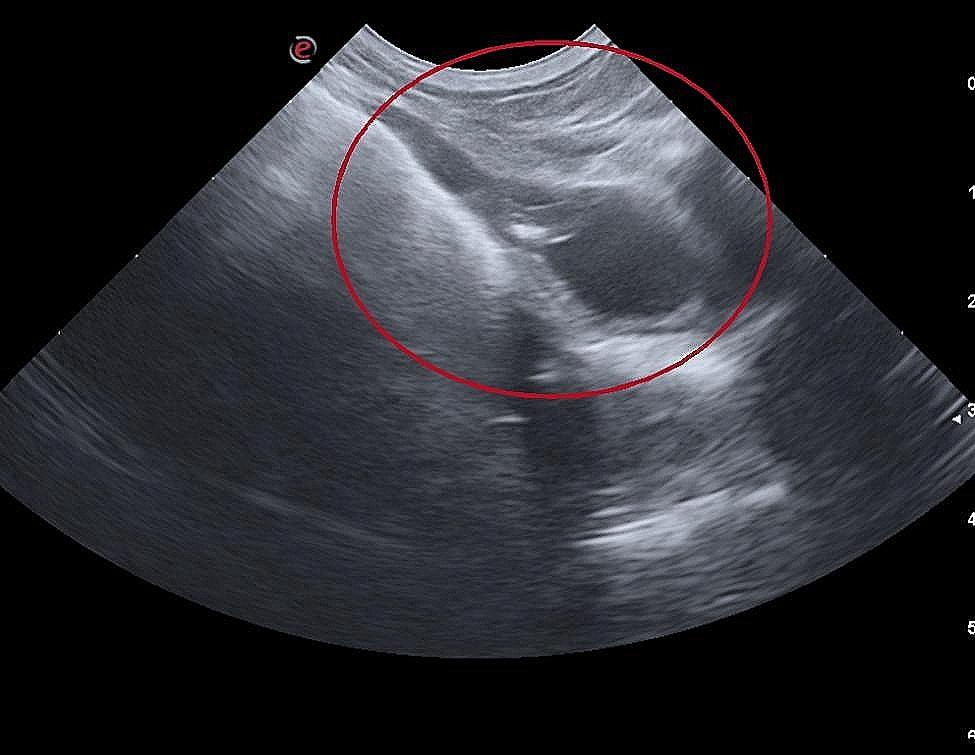
Ultrasonographic examination of the structure found adjacent to the descending colon (red circle)

Abdominal ultrasonography revealed a large amount of stool in the colon, but also a fluid-filled structure, with approximately 1 cm in diameter, adjacent to the descending colon and cranial to the urinary bladder (Fig. 1). The cytological analysis of the content obtained by fine-needle aspiration revealed poor cellularity and rare inflammatory cells (neutrophils and small lymphocytes), which was suggestive of an inflammatory process.

### Computed tomography (CT) findings

CT revealed a tubular structure with a high uptake wall, ventral to the descending colon and caudal to the bladder, which extended cranially, ventral to the bladder, through two branches. Both branches showed a ventrocaudal course; whereas one ended near the colon wall, the other extended towards the left inguinal canal and passed to the inguinal subcutaneous tissue, ending in a nodular structure, with the approximate dimensions of 10 × 9 × 19 mm. The branch that ended caudally, ventral to the colon, presented a cul-de-sac shape and was situated approximately 3 cm from the anus.

**Fig. 2:**
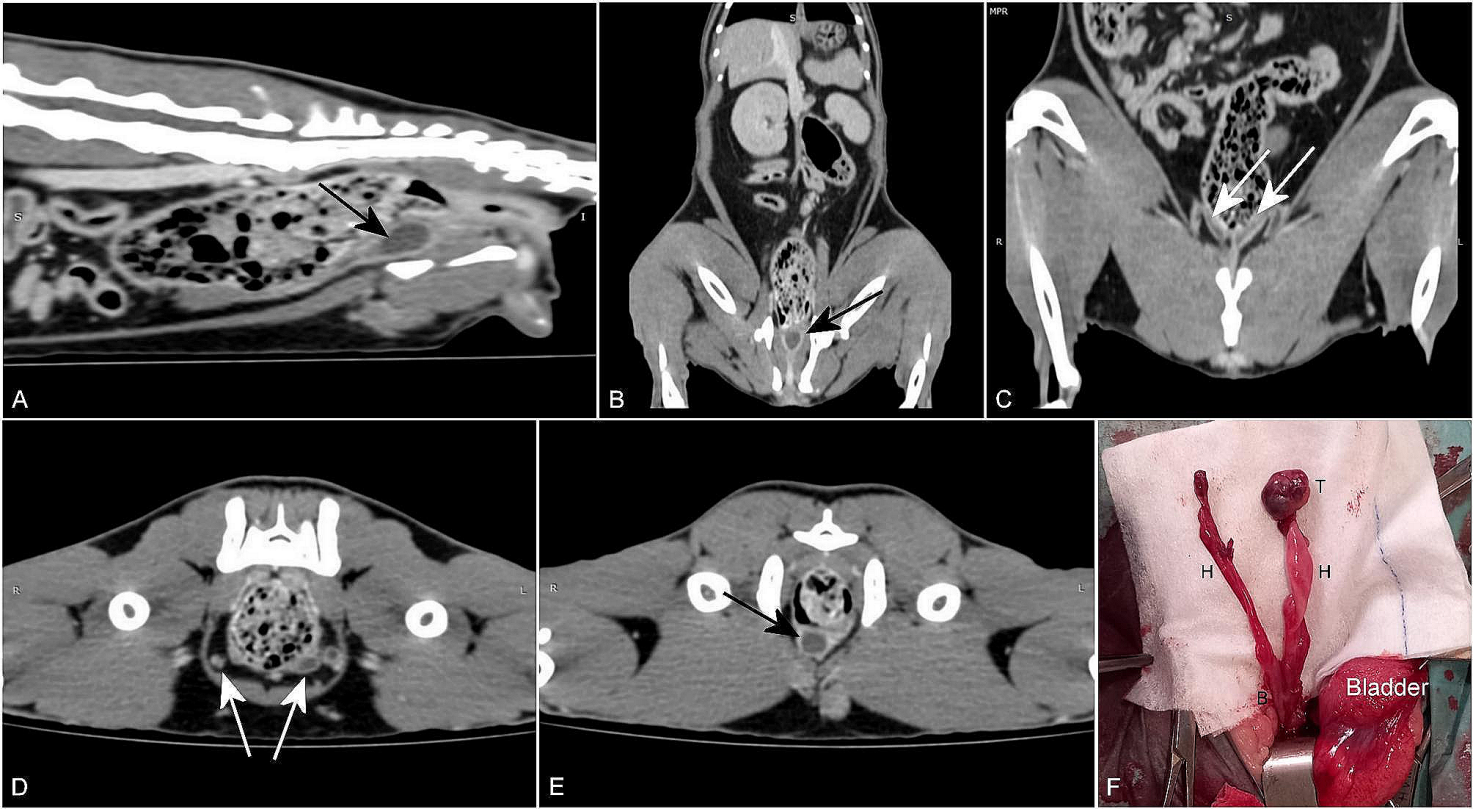
Postcontrast CT images (**A**, **B**, **C**, **D** and **E**) and the gross appearance (**F**) of the anomalous structures identified, adjacent to the descending colon. (**A**) and (**B**) A sagittal and a dorsal CT image, respectively, show a tubular structure with a high uptake wall, ventral to the descending colon (black arrow). (**C**) and (**D**) A dorsal and a transverse CT image, respectively, show the ramification into two branches (white arrows) of the previous identified structure. (**E**) A transverse CT image shows a nodular structure in the left inguinal subcutaneous tissue and a tubular structure, with a high uptake wall and a low uptake interior, ventral to the descending colon, compatible with a remnant uterus (black arrow). (**F**) Intraoperative image of the structures found. The two tubular structures (**H**) converge into a wider structure similar to a uterine body (**B**). At the end of one tubular structure, there is a small structure that resembles a testicle (**T**)

These findings were compatible with vestigial female and male reproductive organs, with what appears to be two uterine horns, a cryptorchid testicle and a tubular structure suspected to be the remnant uterus (Fig. 2A–E).

### Surgery

Exploratory laparotomy was performed and the reproductive tract was removed. The access to the abdominal cavity was made by standard surgical access through midline celiotomy, starting 2 cm caudal to the umbilicus and extended to the pubis. The pelvic cavity was inspected and, at this moment, a tubular structure adhered to the ventral wall of the descending colon and ended in a cul-de-sac with liquid content was identified. With a blunt-tipped scissor, this structure was dissected to its origin, close to the pelvic urethra, where an enlargement of its diameter was verified. From this wider region, another tubular structure raised and which was also dissected. This second structure passed through the left inguinal canal and at its end there was a small structure that resembled a testicle (Fig. 2F). During surgery, a connection between the pelvic urethra and the tubular structure was identified. The structure was removed as near as possible to the pelvic urethra. All tissues removed were submitted to histopathological examination.

### Histology

**Fig. 3:**
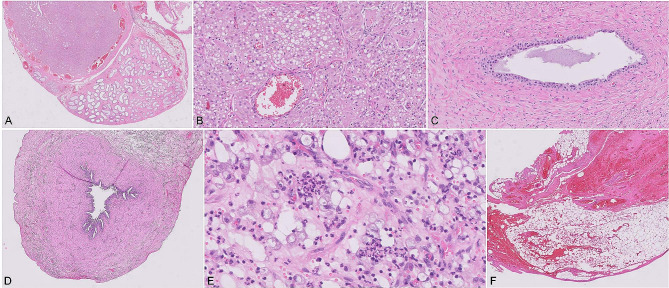
Histopathological findings of the hypoplastic testicle and uterus identified in a domestic Shorthair cat (H&E). Microscopic views of the testicle and epididymis (10×) (**A**), neoplastic area in the testicle (200×) (**B**), vas deferens (200×) (**C**), uterine horn (20×) (**D**), uterine inflammatory infiltrate (400×) (**E**), and fatty tissue with haemorrhage (20×) (**F**)

Histopathological evaluation (Philips^®^ IntelliSite Pathology Solution Ultra Fast Scanner 1.6) evidenced the atrophy of both uterine horns, with normal architecture but poorly developed endometrial glands, and one hypoplastic testicle and epididymis attached to one of its horns (Fig. 3A and B). The testicle exhibited an area composed of neoplastic polyhedral epithelial cells arranged in nests, among rare fibrovascular stroma. These neoplastic cells had finely vacuolated eosinophilic cytoplasm and an ovoid nucleus of moderate pleomorphism. The mitotic index was 1 mitotic figure per 10 high-power fields. These changes are suggestive of Leydig’ cell tumour. Cystic and haemorrhagic areas were also present (Fig. 3B). Continuous with the epididymis there was a vas deferens (Fig. 3C).

The uterine horns (Fig. 3D) were characterized by chronic inflammation (Fig. 3E) with moderated diffuse inflammatory interstitial infiltrate, composed by plasmocytes, lymphocytes, macrophages and a small number of neutrophils.

The end of the other uterine horn was composed by a mass of adipocytes in between the collagenous stroma, with the presence of haemorrhage and vascular cavities (Fig. 3F).

### Cytogenetic analysis

In order to identify a possible genetic background for this case, a cytogenetic analysis in peripheral blood was performed. Cytogenetic evaluation was performed using peripheral blood collected into heparinized tubes. Lymphocyte cultures were established, according to standard protocols [8].

Chromosome preparations were performed by the standard air-drying method, and slides were first stained with 4% Giemsa (Giemsa Merck^®^, Darmstadt, Germany) for detection of numerical chromosome aberrations. Afterwards in order to confirm the results, G-banding protocol was executed. Using an Olympus^®^ CX31 microscope, metaphases were selected with a 100X objective lens and captured using an Olympus EP50 camera. Chromosomes were analyzed by two independent scorers on 60 metaphases, according to ISCN recommendations [9].

**Fig. 4:**
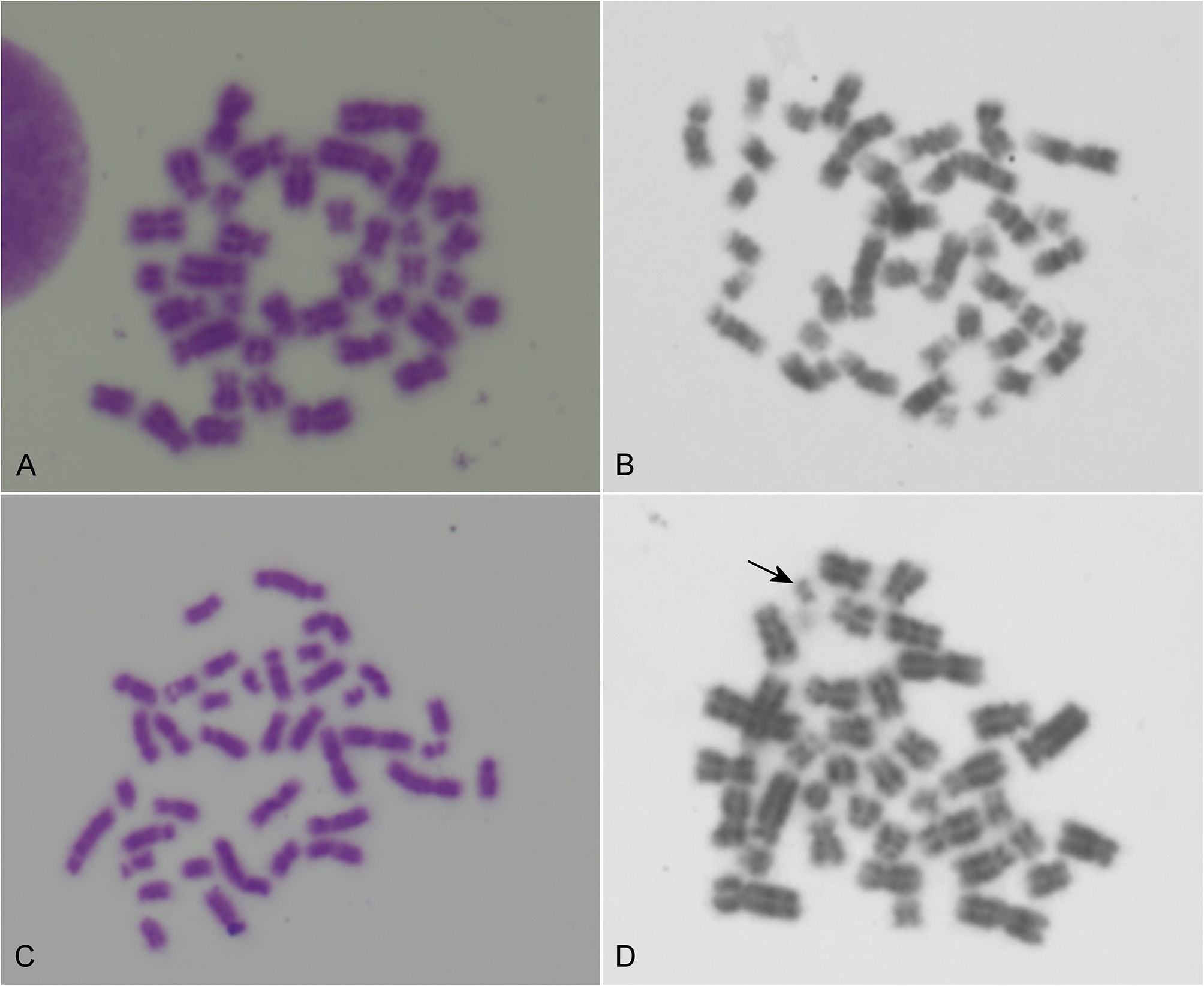
Image representations of the analyzed metaphases. **A** and **B** representative metaphases from the aneuploid 37,X cell line. **C** and **D** representative metaphases from the 38,XY cell line; the arrow indicates the Y chromosome

Results revealed the presence of a mosaic 37,X/38,XY karyotype (Fig. 4). The cell lines were found in 15% and 85% of the lymphocytes, respectively. Fifty-one (85%) metaphases had a karyotype with 38 chromosomes, with presence of X and Y chromosomes, and nine (15%) had karyotype with 37 chromosomes, with absence of the Y chromosome.

### Screening of Y-specific DNA sequences

**Fig. 5:**
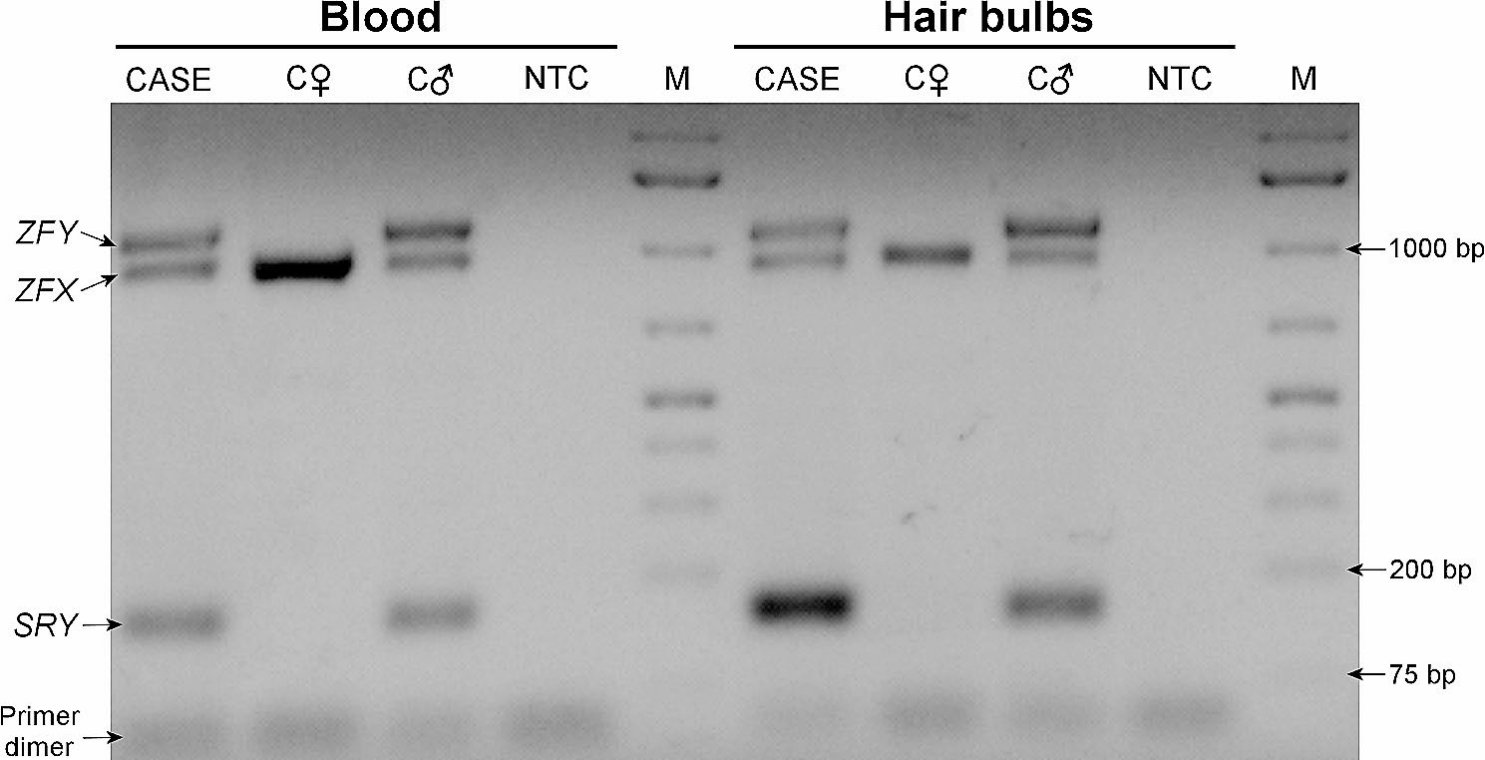
Results of the PCR amplification of *SRY* and *ZFX*/*ZFY* partial sequences. CASE - DSD cat; C♀ - control female cat; C♂ - control male cat; NTC - no-template control; M - GeneRuler 1 kb Plus DNA Ladder (Thermo Fisher Scientific, Waltham, MA, USA)

Samples from whole blood and hair bulbs were collected from the cat under study and two control cats (one male and one female). DNA was extracted using the *Quick*-DNA Miniprep Plus Kit (Zymo Research, Irvine, CA, USA) following the manufacturer’s protocols. The presence of *SRY* and *ZFX*/*ZFY* genes in these cells/tissues were carried out by standard PCR amplification usind primers SRYA-3/SRYB-5 [10] and LGL331/LGL335 [11]. Reactions were performed in multiplex including 5 µl of Supreme NZYTaq II 2x Green Master Mix (NZYtech, Lisbon, Portugal), 2.5 µM of each primer, 2 ul of template DNA (∼ 20 ng) and ultrapure water to make a total volume of 10 ul. The amplification cycle as follows: 95ºC for 5 min, followed by 40 cycles of 95ºC for 30 s, 58ºC for 1 min, 72ºC for 30 s, and a final extension at 60ºC for 10 min. PCR products were separated using 1.5% agarose gel stained with GreenSafe Premium (NZYtech, Lisbon, Portugal). PCR results confirmed the presence of *SRY* and *ZFY* in the DNA isolated from whole blood and hair bulbs of the studied cat (Fig. 5).

### Gene expression analysis

*SRY* (sex-determining region Y gene) is found on the Y chromosome. It is known that induction of *SRY* expression in the bipotential gonad specifies the commencement of testicular differentiation and the sex of the developing individual [12]. Therefore, given the results of cytogenetic analysis performed in peripheral blood, where the majority of cells had a Y chromosome, we analyzed the expression of the *SRY* gene in testicular tissue to better understand the phenotype displayed by the presented cat. For that, histological blocks containing testicular tissue (Fig. 3A) from the cat here reported and from healthy aged-matched control cats were used to extract RNA using the E.Z.N.A FFPE RNA kit (Omega Bio-Tek, Norcross, USA), according to manufacturer instructions. RNA was quantified using the NanoDrop spectrophotometer ND-1000 (Version 3.3; Life Technologies). cDNA conversions were made using the High-Capacity cDNA Reverse Transcription Kits (Applied Biosystems, California, USA). Expression studies were then performed on a Bio-Rad CFX96 (Bio-Rad, Hercules, USA), with amplifications made using NZY qPCR Green (NZYTech, Lisbon, Portugal). *GAPDH* were used as housekeeping gene to normalize gene expression levels. The average CT values of the reference genes were used to calculate the gene expression levels. The feline expression of *SRY* gene (NM_001009240.1) was amplified using forward (ACCTCCAATTACCGGTGTGA) and reverse (GGGGATTCTCTAGAGCCACC) primers, and *GAPDH* (NM_001009307.1) using forward (TCGGTGTGAACGGATTTGGC) and reverse (TTTGCCGTGGGTGGAATCAT) primers. Three control individuals and three technical replicates were performed in each PCR assay and two assays were done. Fold variation of gene expression levels was calculated following a mathematical model using formula 2^−ΔΔCt^ (The Livak Method). Statistical significance was determined using the non-parametric Mann-Whitney test, with the GraphPad Prism software version 9. Significance was set at alpha < 0.05.

**Fig. 6:**
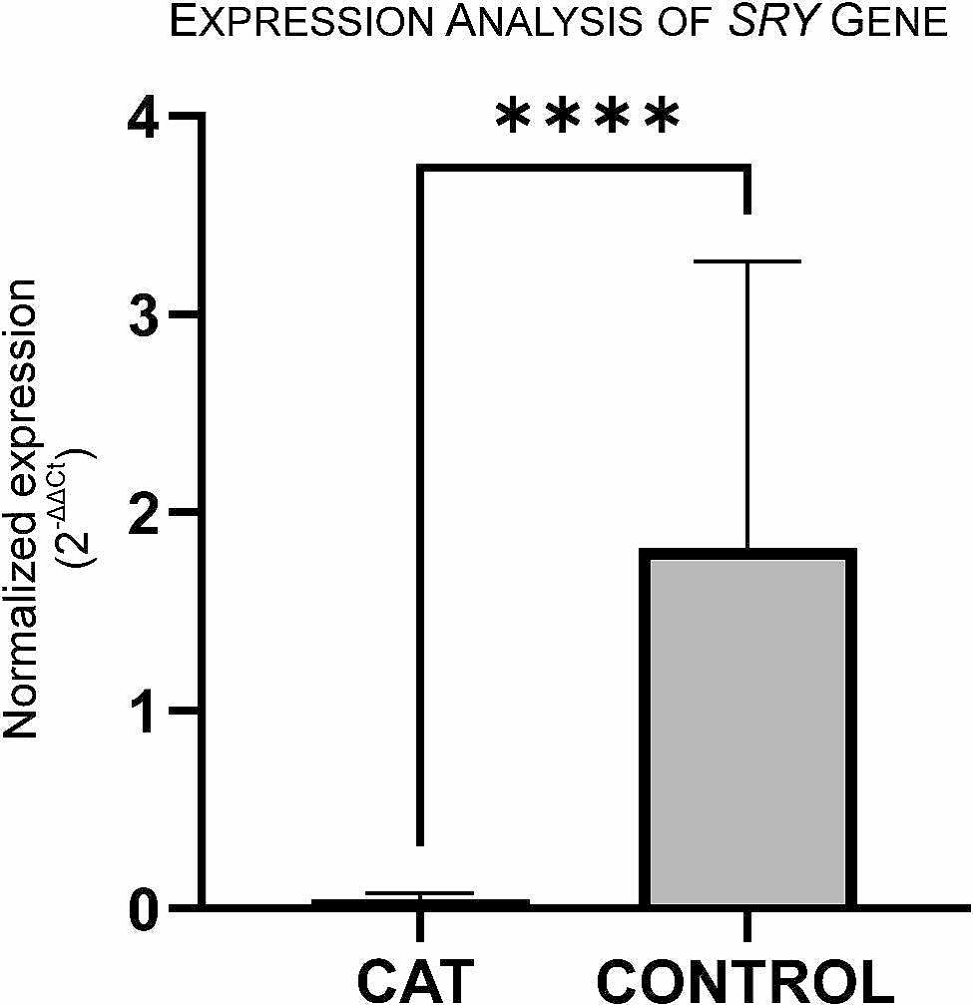
Gene *SRY* mRNA expression levels in testicular tissue from the case here presented (CAT) and control individuals. *GAPDH* were used as housekeeping gene, with results normalized. SYBR Green was the fluorescent dye used. Statistical significance was determined using the Mann-Whitney test. ****p < 0.0001

From the genetic analysis, a 97.5% reduction (log fold change of -1.347) was observed in *SRY* gene expression of the studied cat comparing to control cats (Fig. 6). This reduction of *SRY* expression in comparation to controls may be related to the abnormal sexual development presented by the cat.

## Discussion and conclusions

In males, the sex-determining region of the Y chromosome gene (*SRY*) is responsible for initiate an intricate, interactive cascade of genetic signals. The induction of *SRY* expression in the bipotential gonad specifies the commencement of testicular differentiation and the sex of the developing individual [12].

*SRY* seems to act on the *SOX9* gene activation [13]. In addition to activation by *SRY*, the *SF-1* gene also seems to be necessary to initiate expression of *SOX9*. Both *SRY* and *SF-1* bind in the same initiator sequence region of *SOX9*. *SOX9* then initiates Sertoli cell formation and organization of the sex cords in the embryonic gonad. *SOX9* remains active throughout life inside of the Sertoli cells [5], but even though *SRY* is mainly active during embryogenesis, it is suggested that Spermatogonia, round spermatids, and Sertoli cells of adult testes express *SRY* in various species [14, 15]. The Sertoli cells, under the stimulation of *SF-1*, secrete Müllerian inhibiting substance, which induces the regression of the Müllerian ducts. The Leydig cells, under the influence of the same protein, secrete testosterone, which stimulates the Wolffian ducts to mature into the epididymis and vas deferens [5].

In this work, we describe a rare case of a tomcat that presented a 37,X/38,XY mosaic karyotype in peripheral blood, *SRY*-positive in blood and hair bulbs, and almost complete absence of *SRY* expression in gonads, which allows the inclusion of the case in the group of sex chromosomal DSD. Both, the androgen- and the AMH–dependent masculinization, were likely incomplete in the present case. Several cases of DSD conditions have been described in dogs [16–18], but very little in cats probably due to lack of karyotyping. In one of the few cases described [7], the cat was presented with behavioural problems and visible reproductive concerns, such as cryptorchidism, while in our case, the cat was presented with tenesmus and unspecific clinical signs. CT-scan was crucial in guiding the clinical approach, since none of the clinical signs presented by the animal suggested a connection with the reproductive system. CT-scan identified two tubular structures converging at the pelvic inlet similar to a uterine bifurcation, as described by Balogh et al. [7]. In the last case, two testicles were identified, even though one of them was prescrotal. In the present case, only one cryptorchid testis was present, while in the extremity of the right uterine branch, a structure that although macroscopically resembles a gonad, histologically does not appear to be a streak gonad, since there was not a compact stroma, but a mass of fatty tissue, instead. Considering that the previous history of the animal was not known, our suspicion is that the cat had only the left scrotal testis and may have been previously neutered, at a young age, and returned to its colony until later rescued. In a case previously described [2], the left testicle was found in the scrotal sac, whereas the right one was abdominally located. No behaviour or urinary problems have been reported by the owners. The presence of a hypoplastic testicle and the tumour associated were likely associated to low testosterone levels.

The present clinical case evidenced some unique features, such as the uterine chronic endometritis and Leydig cell tumor in the cryptorchid testicle, which confirmed the rarity of the case. Testicular tumours are rare in domestic cats, with only a few studies on the topic in this species [19]. Cryptorchidism is a risk factor for the development of testicular tumours in dogs, however, this association has not yet been made in cats [20]. The association of the testicular tumour with DSD in mosaic karyotype, to the best of authors’ knowledge, was never described in cats. From a clinical point of view, it should be noted that a complete and exhaustive imaging diagnosis was essential, otherwise the tumour could have developed with metastases.

Findings of the present case are important to understand the diversity of clinical conditions associated to a DSD in cats, generally less described than in dogs. A multidisciplinary diagnostic approach including a complete clinical and imagiological workup and genetic analysis should be considered in each DSD suspicion, in order to correctly classify these conditions in cats.

## Data Availability

All data generated or analysed during this study are included in this published article.

## Abbreviations

DSD: Disorder of Sexual Development
SRY: Sex Determining Region Y
ZFY: Zinc Finger Protein Y-Linked
ZFX: Zinc Finger Protein X-Linked
CT: Computed tomography
ISCN: International System for Human Cytogenomic Nomenclature
PCR: Polymerase chain reaction
NTC: No-template control
Ct: Cycle threshold
GAPDH: Glyceraldehyde-3-Phosphate Dehydrogenase
SOX9: SRY-Box Transcription Factor 9
SF-1: Steroidogenic Factor 1
AMH: Anti-Müllerian hormone
